# Pentraxin 3: A Main Driver of Inflammation and Immune System Dysfunction in the Tumor Microenvironment of Glioblastoma

**DOI:** 10.3390/cancers16091637

**Published:** 2024-04-24

**Authors:** Sarah Adriana Scuderi, Alessio Ardizzone, Ayomide Eniola Salako, Giuseppe Pantò, Fabiola De Luca, Emanuela Esposito, Anna Paola Capra

**Affiliations:** 1 Department of Chemical, Biological, Pharmaceutical and Environmental Sciences, University of Messina, Viale Ferdinando Stagno D’Alcontres, 31, 98166 Messina, Italy; sarahadriana.scuderi@unime.it (S.A.S.); aleardizzone@unime.it (A.A.); ayomide.salako@studenti.unime.it (A.E.S.); fabiola.deluca@unime.it (F.D.L.); annapaola.capra@unime.it (A.P.C.); 2 University of Florence, 50121 Florence, Italy; 3 Department of Biomedical and Dental Sciences and Morphofunctional Imaging, University of Messina, Via Consolare Valeria 1, 98125 Messina, Italy; peppepanto@hotmail.it

**Keywords:** glioblastoma, pentraxin 3, immunotherapy, novel biomarkers, inflammation, immune system

## Abstract

**Simple Summary:**

Brain tumors, including glioblastoma (GB), pose a significant health concern globally, with high mortality rates despite current therapies. Pentraxin 3 (PTX3) is a multifunctional regulatory protein implicated from the early stages of inflammatory processes. Recent studies suggest PTX3’s involvement in tumor progression, including metastasis and invasion, in multiple cancer types. However, the role of PTX3 in GB remains poorly understood. This review aims to discuss PTX3’s function in GB pathology, considering its diverse biological activities and its potential as a promising molecular target. Understanding PTX3’s role in GB could provide insights into novel therapeutic approaches to improve patient outcomes and survival rates.

**Abstract:**

Brain tumors are a heterogeneous group of brain neoplasms that are highly prevalent in individuals of all ages worldwide. Within this pathological framework, the most prevalent and aggressive type of primary brain tumor is glioblastoma (GB), a subtype of glioma that falls within the IV-grade astrocytoma group. The death rate for patients with GB remains high, occurring within a few months after diagnosis, even with the gold-standard therapies now available, such as surgery, radiation, or a pharmaceutical approach with Temozolomide. For this reason, it is crucial to continue looking for cutting-edge therapeutic options to raise patients’ survival chances. Pentraxin 3 (PTX3) is a multifunctional protein that has a variety of regulatory roles in inflammatory processes related to extracellular matrix (ECM). An increase in PTX3 blood levels is considered a trustworthy factor associated with the beginning of inflammation. Moreover, scientific evidence suggested that PTX3 is a sensitive and earlier inflammation-related marker compared to the short pentraxin C-reactive protein (CRP). In several tumoral subtypes, via regulating complement-dependent and macrophage-associated tumor-promoting inflammation, it has been demonstrated that PTX3 may function as a promoter of cancer metastasis, invasion, and stemness. Our review aims to deeply evaluate the function of PTX3 in the pathological context of GB, considering its pivotal biological activities and its possible role as a molecular target for future therapies.

## 1. Introduction

Glioblastoma (GB) is a difficult-to-treat, extensively lethal, aggressive intracranial malignancy that is the most common primary brain tumor [1]. The standard therapy options currently include resection, radiation, and chemotherapy using several drugs [2,3,4]. Despite this multimodal approach, the median survival rate is still approximately 14 months [2,3,4]. GB is referred to as a multiforme because of the heterogeneous nature of the tumor in terms of its regressive response to treatment, clinical manifestations, multiple molecular biomarkers, and a very complex physiopathology. Additionally, GB genetic and chromosomal aberrations largely contribute to its invasive nature. Another important clinical challenge in the context of GB is the blood–brain barrier (BBB), which mechanically restricts the passage of the drug molecules and consequent delivery of the chemotherapeutic agents to the aimed targets [5,6].

The GB tumor microenvironment has multiple cell types including microglia, astrocytes, fibroblasts, vascular cells, and macrophages cell types that form a neurovascular unit function strongly contributing to tumor progression [7,8]. As an immunologically quiet tumor, GB has a low tumor mutational burden, and few tumors infiltrating T cells, compared to other tumors, that have had success with immunotherapy [9,10]. Checkpoint inhibition, such as Programmed Cell Death 1 (PD-1), Programmed Cell Death Ligand 1 (PD-L1), and cytotoxic T lymphocyte-associated protein 4 (CTLA-4), have been one of the most prominent targets in several advanced malignancies [11]. Even though preclinical data from certain studies show prospects of checkpoint inhibitors for GB treatment, there is still the challenge of central nervous system (CNS) toxicity [12,13].

Alongside these trials, there are ongoing studies dedicated to understanding how to use biomarkers to respond to checkpoint inhibitors. These studies have probed the ability of biomarkers to predict response to PD1 and PD-L1 inhibitors in a study evaluating 94 GB patients. The authors found median PD-L1 expression to be 2.77%, and that PD-L1 expression correlated with worse outcome [14], while previous data did not find PD-L1 to be a negative prognostic factor [15]. These contrasting findings make the role of PD-L1 expression on GB tumor cells in response to checkpoint inhibition unclear. In another study with preclinical results, the adenosinergic pathway (AP) represents another major player in the suppression of antitumor immune responses in the GB tumor microenvironment (TME); the suppression occurs through the signaling of adenosine receptors on immune cells overriding the chemoresistance [16].

In this frame, the search for novel molecular predictors represents a crucial objective to improve diagnosis, therapies, and prognosis in oncology [17].

Pentraxin 3 (PTX 3) belongs to the pentraxin superfamily, a superfamily of proteins that share the same domain [18], and is identified by a 205 amino acids long conserved sequence at the C-terminal [19,20]. The pentraxin superfamily is divided into two major subgroups: the short, and the long pentraxin. Long pentraxins are about twice the size of short pentraxins, comprising neuronal pentraxin 1 and 2 (NPTX1, NPTX2), pentraxin 3 and 4 (PTX3 and PTX4) [21], are soluble pattern recognition molecules (PRMs) and have the ability to mediate humoral host response, and are prominent as a major player in innate immunity and inflammation [20,22], while short pentraxins are composed of C-reactive protein (CRP) and serum amyloid P component (SAP) [21].

The pentraxin superfamily have been implicated with the ability to interact with the extracellular matrix (ECM) proteins, modifying the structures of deprecating host cells to maintain homeostasis [23]. Most of the members can also activate the PI3K/AKT/mTOR pathways, which implies interference with the normal cell cycle, with PTX3 particularly identified to have unique receptors that mediate tumor progression [24]. Additionally, pentraxins can mediate phagocytosis and inflammation by interacting with the surface Fcγ receptors upon target opsonization, important for innate immune defense [25].

In several studies [26,27], investigations into pathological conditions with inflammatory/infectious origins have confirmed a direct correlation with the rapid rise of PTX3 blood levels, especially in humans and mice [28]. An increase in the synthesis of inflammatory biomarkers and consequent activation of different cellular mechanisms have been confirmed to induce PTX3 expressions in different cells, including neutrophils, monocytes, lymphocytes, fibroblasts, and epithelial cells [29,30]. Moreover, PTX3 shows oncogenic dual function abilities, both pro- and anti-tumor functions [20].

It has been confirmed that PTX3 Is associated with many non-malignant conditions that include stroke, sepsis, hepatic cirrhosis, systemic lupus, atherosclerosis, and rheumatoid arthritis, with eventual overexpression in different types of tumoral conditions including breast, lung, prostate, gliomas, and hepatic cancer [31,32,33,34].

In the TME of gliomas, PTX3 has been reported to be an important part as it is secreted and expressed by tumor cells where tumor-associated macrophages (TAM) are the dominant factor [35,36], while in GB, previous research has implicated PTX3 to be closely related to the expression of PD-1, PD-L1, CD276, and HAVCR2 in the TEM, with the expression of PTX3 significantly increasing with the worsening prognosis of GB [36]. Specifically, PTX3 has been found to be active in the critical modulation of the immunosuppressive microenvironment, and in the mediation of macrophages infiltration, migration, and inflammation-resolving polarization [36].

There is sufficient evidence to suggest that PTX3 is a biomarker with immunotherapeutic checkpoint inhibitors, and which has the possibility of countering the immunosuppressive effects of chemotherapeutic agents [20]. Additionally, an increase in PTX3 blood levels has been identified as a reliable biomarker of the start of inflammation [28].

Based on all these suggestions, this review aims to explore the function of PTX3 as a core driver of immunity and inflammation in GB, deeply investigating its biological activities, especially within the TME, and its role as a potential target for future GB immunotherapeutic efforts.

## 2. Primary Brain Tumors Background: Focus on Glioblastoma

Primary brain tumors develop from the tissues of the brain or the brain’s immediate surroundings, involving several cell types [37]. The classification of brain tumors is based on their histological, physiological, genetic, and molecular characteristics [38]. Primary brain tumors can be classified in glial and non-glial tumors, and in benign (non-cancerous) or malignant (cancerous) tumors. The glial tumors originate from glial cells while the non-glial tumors develop on or in the structures of the brain, including nerves, blood vessels, and glands. Primary brain tumors are characterized by nuclear atypia, mitotic activity, endothelial proliferation, necrosis, and high invasiveness [38]. The complexity of the different types of brain tumors and their prognosis depends on their origin, their development, and progression [38]. Therefore, it is extremely important that the origin, which involves the formation of cancer stem/progenitor cells, is explored to understand the growth, prognosis, treatments, drug resistance, and relapse of brain tumors [38].

In the last decade, more than 150 different brain tumors have been identified [39]. However, in this review, we focused on GB as the most common and aggressive CNS tumor.

Gliomas are malignant brain tumors that originate from glial cells surrounding brain tissue. The incidence of glioma is about 8 per 100,000 people worldwide, and it increases with age [40]. Gliomas are classified into: pilocytic astrocytomas (Grade I), which are benign tumors and occur primarily in children; astrocytomas, oligodendrogliomas, and oligoastrocytomas, which correspond to low-grade (II) or high-grade (III and IV) and can develop into GB, a grade IV astrocytoma [41]. GB is the most aggressive primary tumor of the CNS, accounting for 48.6% of all malignant CNS tumors [41]. GB diagnosis is associated with a median survival of approximately 15 months and a 5-year survival of approximately 36% [42]. GB arises from glial cells with a surrounding brain parenchyma comprising CNS-specific cells including astrocytes, neurons, and microglia, and a distinctive ECM composition [43].

According to the World Health Organization (WHO), GB is classified into:

- Isocitrate dehydrogenase (IDH)-wild type GB (about 90% of cases), which corresponds to the clinically defined primary or de novo tumor. It develops without evidence of a malignant precursor. It is prevalent in patients aged over 55 years [44].
- IDH-mutant GB (about 10% of cases), which corresponds to the secondary GB. It develops from low-grade diffuse astrocytoma (WHO grade II diffuse astrocytoma) or anaplastic astrocytoma (Grade III), and occurs in younger patients (mean age = 40 years) [44].
- Not-otherwise-specified GB (NOS), which is reserved for those tumors for which full IDH evaluation cannot be performed [44].
- Not-elsewhere-classified GB (NEC) occurs because of discrepancies between the clinical, histological, immunohistological, and genetic tumor features [44].

Currently, the standard treatment for GB consists of surgical resection and adjuvant chemotherapy with temozolomide (TMZ), a pro-drug alkylating agent, combined with radiotherapy [45]. However, despite the progress in conventional treatments, the outcome for patients with GB remains almost fatal due to the therapeutic resistance and tumor recurrence after surgery [46].

Thus, comprehending GB pathogenesis is necessary for both the identification of novel biomarkers, and the design and development of potential chemotherapeutic treatments.

Basically, cancer develops in part from a failure of normal immune surveillance [47]. Over the last decade, it has been demonstrated that both innate and adaptive immunity play an important role in counteracting and facilitating cancer development, as well as in contributing to standard therapies [48]. The GB microenvironment is heterogeneous; it involves neoplastic, but also non-neoplastic cells, including infiltrating and resident immune cells, vascular cells, and other glial cells [49]. Multiple studies have demonstrated the immunosuppressive nature of glioma [47,50]. In particular, research focused on the role of tumor-associated macrophages (TAMs) in GB [51], which originate from two independent sources: brain-resident microglia, and/or bone marrow-derived monocytes [52]. In non-pathological settings, microglia represent the main innate immune cells in the brain as mechanisms of defense against pathogens and harmful stimuli. However, in the context of tumors, microglial cells contribute to initiating and maintaining tumor growth and spread [53]. TAMs in brain cancers are the most abundant population of immune cells, accounting for up to 50% of total live cells in the whole GBM tumor mass [54]. TAMs have been shown to reciprocally interact with tumor cells, promoting tumor growth and progression. The recruitment of TAMs is mostly mediated by cytokines and chemokines released by GB cells, which include CC-chemokine ligand 2 (CCL2; also known as MCP1) and CCL7 (also known as MCP3), glial cell line-derived neurotrophic factor (GDNF), hepatocyte growth factor (HGF), SDF1, tumor necrosis factor (TNF), vascular endothelial growth factor (VEGF), adenosine triphosphate (ATP), macrophage colony-stimulating factor 1 (CSF1), and granulocyte–macrophage colony-stimulating factor (GM–CSF), which consequently regulate antitumor immune response [55]. Several studies have confirmed larger numbers of TAMs in higher-grade gliomas compared with lower-grade tumors [56,57].

GB tumor cells may downregulate major histocompatibility complex (MHC) molecules expression to avoid neoantigen presentation [58]. According to this, it has been described a downregulation of MHC class II molecules, which are essential for cross-presentation of antigens to adaptive immune cells in GB, underscoring the broader immunosuppressive effects of the tumor [58].

Glioma cells express increased levels of immunosuppressive factors such as PD-L1 and indolamine 2,3-dioxygenase (IDO) [59]. In the tumor microenvironment, PD-1, a cell surface receptor expressed on a variety of immune cells, and its ligand PD-L1, expressed in tumor cells and antigen-presenting cells (APCs), play a critical role in tumor progression and survival by escaping immune surveillance [60]. In the context of glioma, PD-L1 has been considerably studied to be related to cancer progression by suppressing the activity of T lymphocytes and mediating immune evasion by cancer cells [61]. GB cells can secrete several immunosuppressive chemokines and cytokines, such as interleukin (IL)-6, IL-10, transforming growth factor (TGF)-β, galectin-1, and prostaglandin-E, which act on infiltrating immune cells to hijack them by inducing a protumor cellular phenotype [62]. The GB microenvironment, particularly through the release of IL-6 and the expression of PD-L1 and IDO-1, is able to promote the formation of regulatory T cells (Tregs) that blunt the anti-tumor T cell response [63,64]. As a result, Tregs release the immunosuppressive cytokine IL-10, which inhibits T cell proliferation and blocks the anti-tumor immune responses, attenuating T cell cytotoxic activity.

The release of dysregulated levels of cytokines and growth factors by glioma stem cells (GSC) and immune cells within the GB microenvironment can potentially enhance invasive and angiogenic processes, contributing to tumor growth [65,66].

Currently, several aspects contribute to making gliomas difficult to treat, such as the anatomical location, the presence of the BBB, which hinders the delivery of therapeutic compounds to the tumor site, and the limited immune reactivity within the CNS [67].

In recent years, new immunotherapeutic strategies have been well-studied in various preclinical and clinical studies [68,69,70,71]. Previous research has shown that therapeutic inhibition of PD-L1 or IDO in glioma mouse models decreases tumor-infiltrating Treg cell numbers and significantly increases long-term survival [71]. In an in vivo orthotopic study, researchers found that the blockade of PD-1 could promote the cytotoxicity of NK cells against GSC, suggesting that immunostimulatory checkpoint inhibitors (ICI) could be an effective strategy for treating GB [69].

Furthermore, in recent years, the efficacy of CAR-T therapy in glioma has been proved [68,72,73]. Chimeric antigen receptors (CARs) are genetically synthetic immunoglobulin T cell receptor molecules that can recognize specific antigens and activate T cells [74]. Since CAR-T cell therapy opened the door to a new era of cancer treatment, multiple CARs have been developed to treat glioma, including CARs targeting IL13Rα2, EGFRvIII, and CD70 [68,72]. In this regard, a previous study showed the promising antitumor activity of first-generation IL13Rα2 CAR-T cells in patients with GB [68]. Currently, 26 clinical trials using CAR-T cells in the treatment of glioma have been registered [56].

Moreover, immunotherapy by dendritic cells (DCs) has gained great attention as a potential approach to enhance anti-tumor immunity in gliomas. As the “professional” antigen processing and presenting cells, DCs play a crucial role in the initiation of the anti-tumor immune response [75]. Several pre-clinical studies have revealed long-term tumor survival and increased immunological memory in murine models of GB through the stimulation of DCs activity with various antigens and costimulatory molecules [75,76]. These advances have inspired great interest in clinical immunotherapeutic trials of DC-based vaccines against GB.

Despite these scientific advances, we must consider that GB is characterized by high molecular heterogeneity, which may limit the efficacy of the treatment by evading the targeted immune response; for this purpose, further studies are needed.

## 3. Function of Innate Immunity in Cancer and Related Therapeutic Strategies

As stated, accumulating gene mutations and structural changes promote malignant transformation and increase the immunogenicity of cancer cells along the course of cancer evolution [77].

The host immune system may identify tumor antigens produced by altered genes as non-self and start the immune system’s removal [78]. Tumor antigen capture is the first stage in the cascade multi-step process that leads to immune death in immune-mediated elimination, which is coordinated by innate immunity and adaptive immunity [79,80]. With its combination of chemical and physical barriers, as well as different kinds of immune cells equipped with pattern-recognition receptors (PRRs), innate immunity functions as the first line of protection for the host.

Adaptive immune response and non-specific killing of malignant cells are the main ways that innate immune components, which include dendritic cells (DCs), macrophages, monocytes, neutrophils, eosinophils, basophils, mast cells, natural killer (NK) cells, natural killer T (NKT) cells, γδ T cells, and mucosa-associated invariant T (MAIT) cells, slow tumor growth [81]. Unlike the innate arm, the adaptive arm of host immunity uses T and B cells to attack cancer cells [82].

All altered cells should ideally be identified by host immunity and destroyed; nonetheless, cancer is a heterogeneous illness, with several subclones exhibiting an unequal distribution of extensive genetic and epigenetic changes [83,84]. Tumor subclones with low immunogenicity can evade immune-mediated tumor clearance when adaptive immunity acts as a selection pressure [85]. Selected subclones are supported when developing into clinically evident lesions by their poor immunogenicity in conjunction with immunosuppressive mechanisms, including immune checkpoint pathways, metabolite reprogramming, and dysregulated cytokine repertoire [86,87,88].

TAMs, regulatory T (Treg) cells, regulatory B (Breg) cells, myeloid-derived suppressor cells (MDSCs), tumor-associated neutrophils (TANs), and cancer-associated fibroblasts (CAFs) are among the immunosuppressive cell populations in the tumor microenvironment (TME) that also aid in immune evasion and the advancement of cancer [89,90,91]

Consequently, cancer immunotherapies have been reexamined and acknowledged as the fourth therapeutic approach, following radiation, chemotherapy, and surgery.

Immunotherapy is the use of techniques that can alter immune system functioning by influencing the humoral and cellular systems that control the body’s reaction to an antigen. Immunotherapy has revolutionized the area of oncology by strengthening the body’s natural defenses to eradicate cancer cells. Cancer immunotherapy seeks to modulate the immune system without inducing uncontrollable autoimmune inflammatory reactions that can result in therapeutic constraints. Immunotherapies can be broadly divided into two categories: active, and passive [92]. As in the case of oncolytic vaccinations, active immunotherapy refers to the direct induction of an immune response, immunological memory, and a durable response [93]. Both a specific and non-specific response may result from such active vaccination.

The non-specific response happens when the intention is to produce an adaptable host response to combat cancerous cells. A specific response happens when an antigen or, more accurately, an immunogen, is administered to produce an antibody, cytotoxic T lymphocytes (CTL), or a combination of responses against an antigen linked to a particular tumor [94]. Passive immunotherapy, on the other hand, uses a reactive lymphocyte that may identify the malignant tumor cell or an antibody against one or more specifically defined antigens [92].

When the objective is to trigger the host’s adaptive response, this process can generate either non-specific or specific responses. The latter is produced when the target is directed against a specific malignant cell, but as is the case with monoclonal antibodies, these responses are transient and may need ongoing therapy [95]. While immunotherapeutic techniques have demonstrated remarkable efficacy in treating a variety of tumor forms, its use in patients with primary brain tumors has yielded subpar survival rates to date [96]; this is because brain tissue is distinguished by distinct cell types, and the BBB plays a role in making the brain a relatively immune-privileged organ. Highly controlled immune responses in immune-privileged organs lead to an inherently immunosuppressive milieu. Moreover, poor immunogenicity and immunosuppression are also caused by some inherent features of primary brain tumors, such as their high degree of heterogeneity [97].

Immune checkpoint blockage [98] and adoptive cell transfer [99,100] are two examples of antitumor immunotherapies that have received widespread validation and clinical approval for treating a variety of malignancies through the facilitation of T-cell-mediated antitumor responses.

Immune checkpoint molecules, which suppress T cell receptor (TCR) signaling or weaken the costimulatory pathway, are frequently overexpressed in the TME and impede T cell activation [101,102]. Immune checkpoint antibodies disrupt T cell immunosuppressive pathways, including cytotoxic T lymphocyte-associated protein 4 (CTLA-4)-CD80/CD86 signaling and PD-1 and PD-L1 [103,104]. Over 10 anti-PD-1/PD-L1 antibodies have been licensed for use in the treatment of cancer thus far.

Even though these immunotherapies have been incredibly successful in treating advanced tumors, there are still a few challenges that need to be overcome, such as the poor response rate and the absence of reliable biomarkers [105]. Immune checkpoint inhibition was thought to be appropriate for 43.63% of all cancer patients [106], and in the US, the total response rate was less than 13% [107].

### The Link between the Immune System and GB

There has been debate on the brain’s accessibility to immunotherapy and the afferent and efferent arms of the immune system for many years. In fact, Tumor-infiltrating lymphocytes (TILs) and other immune effector cell types are less common in CNS tumors than in other tumor types [108]. Immunostimulatory treatments like immunological checkpoint are linked to suboptimal results in patients with this “cold tumor” characteristic.

Antigen-specific TIL counts can be relatively low, even when T cell responses to CNS tumors are triggered by methods like immunization, and those cells that are present often have an exhausted phenotype [109]. The distinct immunological milieu of the brain is primarily responsible for the decreased number and restricted activation of T cells in CNS tumors [110]. Unrestrained inflammation in the brain presents a concern not often observed in peripheral organs because of its solid enclosure and the possibility of injury from increasing intracranial pressure. Because of this, it is possible that the CNS has developed to be a setting where inflammatory and adaptive immune responses are closely controlled.

Numerous immunosuppressive mechanisms operate at the molecular and cellular levels in this regulation [111]. Brain stromal cells secrete a significant amount of transforming growth factor β (TGF-β) and IL-10, two classic immunosuppressive cytokines that counteract inflammatory cytokines to preserve homeostasis, in response to inflammatory stimuli, including those produced from tumors [112]. Large quantities of indolamine 2,3-dioxygenase (IDO) are produced by glioma cells. IDO depletes the microenvironment of tryptophan, which reduces T cell activity and promotes the formation of regulatory T (Treg) cells [113]. Likewise, high amounts of arginase are produced by both tumor-infiltrating myeloid cells and microglia, which limits T cell proliferation and function by lowering tissue arginine levels [114].

Specifically focusing on GB, it has been found that GB cells create and sustain this environment which suppresses the immune system [115]. Indeed, both TGF-β and IL-10, which reduce inflammation, are produced by these cells. These proteins impair the potency of antigen-presenting cells and reduce the reactivity of specific T lymphocytes. Conversely, they increase Tregs’ population size and impact. Tregs are also drawn to the tumor site by substances released by glioblastoma cells, such as CCL2 [115]. Furthermore, the immunological cell composition of the glioblastoma microenvironment is characterized by a deficiency of lymphocytes and a surplus of certain myeloid cells and macrophages. Because they help in the creation of new blood vessels and block the body’s adaptive immune response, TAMs contribute to GB progression [116].

An essential component of GB’s immune-suppressive milieu is the myeloid-derived suppressor cell (MDSC), a heterogeneous cell population. Through their interaction with many cell types within the tumor, these cells impede immune responses. Their many roles include enhancing the function of regulatory T cells, inhibiting the presentation of antigens, and limiting the activity of specific T cells. Significantly, it has been shown that myeloid cells such as MDSCs and TAMs in glioblastoma patients generate higher amounts of a protein known as programmed death-ligand 1 (PD-L1), which has an adverse effect on immune responses. This further reduces the body’s natural defenses and adds to the disruption of T cell function [117].

By boosting certain proteins that reduce the immune response, the use of inhibitory checkpoints, for example, might ensure protection in GB. Even against more advanced tumors, blocking CTLA-4 and PD-1 combined demonstrated remarkable efficacy in GB-afflicted animals [118]. These strategies are being investigated in clinical trials for GBM that has just been diagnosed or that is recurring. However, the outcomes for GB, especially when combined with conventional therapy, have not been as encouraging as those for other malignancies [119].

For example, in a study named CheckMate-143, nivolumab (anti-PD-1) and bevacizumab were evaluated in patients with recurrent GB, although the two therapies did not significantly vary in overall survival [120]. Moreover, a comparable trial called CheckMate-498 was discontinued when newly diagnosed GB patients without a particular gene change did not exhibit improved survival with nivolumab with radiation than with temozolomide plus radiation [116]. A different experiment called CheckMate 548 did not demonstrate an improvement in median overall survival in patients with O6-methylguanine-DNA methyltransferase (MGMT)-methylated recurrent GB. This trial assessed nivolumab in conjunction with or without radiation treatment and TMZ [118].

Nevertheless, a recent study by Cloughesy et al. showed that patients with recurrent glioblastoma who received adjuvant therapy following surgery and neoadjuvant treatment with pembrolizumab (anti-PD-1) had a significantly better overall survival than patients who only received adjuvant post-surgical pembrolizumab treatment [121]. Apart from CTLA-4 and PD-1/PD-L1 treatments, scientists are investigating alternative checkpoint targets such as CD39 and TIM-3. Similar to PD-1, TIM-3, which is found on other immune cells, can cause T cell fatigue. It has come to light that the ectonucleotidase CD39 inhibits antitumor immunity [118]. Using checkpoint inhibitors in conjunction with other immune-suppressive therapies might be an improvement. It is important to be aware that checkpoint inhibitors may have serious adverse effects in the CNS. There is a real fear that the brain’s too robust immune response might have negative consequences.

In summary, while immunotherapies such as checkpoint inhibitors have shown promise, their role in treating GB is still being determined. More research is needed to determine the best combinations of treatments and to learn more about the immunological and genetic components of GB tumors. Later on, employing checkpoint inhibitors successfully will depend on your understanding of this.

Thus, the search for new molecular biomarkers and targets that drive the immune system is crucial for cancer research, and, in this regard, PTX3 can represent possible candidates.

## 4. Pentraxin 3

The pentraxin superfamily, which includes PTX3, is a group of proteins that have been conserved throughout evolution and play crucial roles in the detection of both self and non-self antigens [18,122].

PTX3 is rapidly produced by various cell types, including myeloid cells, endothelial cells, and respiratory epithelial cells [18,122].

PTX3 has a role in several physiological processes, including complement system regulation, inflammation, immune cell recruitment, and tissue repair [123]. However, PTX3 appears to be implicated in the development of pathological inflammatory processes, which is at odds with its physiological role [124]. PTX3 plays several roles in the organism, one of which is pathogen identification and removal through its function as an opsonin, which in turn promotes phagocytosis [125].

PTX3 has been identified as a crucial factor involved in humoral innate immunity, contributing to both the control of inflammation and tolerance to certain infections [126].

The resemblance between PTX3 and short pentraxin C-reactive protein (CRP) led to studies on the potential use of PTX3 as a marker in a range of infectious and inflammatory human diseases. A pro-inflammatory insult can cause a considerable rise in PTX3 levels as early as 6 h; instead, CRP levels require 24 to 30 h to increase [127], and it is primarily produced by the liver in response to interleukin (IL)-6 during the acute phase response [128]. Differently, tissue cells and serum leukocytes, such as fibroblasts, endothelial cells, monocytes, macrophages, and dendritic cells, can swiftly produce PTX3 in response to various stimuli, including TNF-α and IL-1, PAMPs, and HDL [23,129].

The fast rise of PTX3 in these settings is explained by the release of the preformed protein by neutrophils in response to initial pro-inflammatory cytokines or microbial identification, as well as local synthesis by other cell types at inflammatory sites.

Complement receptor 3 (CD11b/CD18) interaction is the primary mechanism via which the alternative complement pathway is activated [127]. In addition to this, through a calcium-independent method, PTX3 can interact with complement component 1q (C1q) [130], activating the classical complement pathway in the process. Therefore, whereas PTX3 may aid in the removal of pathogens, it may also make pathological inflammatory responses more severe [124].

Upon release, PTX3 impacts several receptors implicated in different aspects of the inflammatory response, including angiogenesis and tissue repair mechanisms [131]. Focusing on its function in inflammation and the immunological response, one of PTX3’s most well-characterized effector mechanisms may be seen in its complex interactions with elements of several complement pathways [132]. Data from scientific studies indicate that PTX3 has the direct ability to increase TLR4-mediated NF-kB signaling, which is a central mechanism for the stimulation of several pro-inflammatory genes and the release of inflammatory mediators [33]. Lastly, research has demonstrated that PTX3 can influence the adhesion molecule P-selectin using its glycosidic domain, which in turn controls the recruitment of inflammatory cells [126].

Through a mechanism mediated by a complement system, PTX3 can increase and extend the inflammatory response by stimulating neutrophil phagocytic activity [127]. Furthermore, PTX3 interacts with the lectin route by networking with mannose-binding lectin, ficolin-1, and ficolin-2 [132].

In addition to the complement system’s activation, several other receptors implicated in the immune response have been discovered recently. By dramatically increasing the neutrophil response, PTX3 prolongs and intensifies the inflammatory process [127]. Additionally, high plasma concentrations of PTX3 have been linked to mortality and illness severity in a variety of clinical diseases [122], thus supporting the suggestion that this pentraxin functions as a biomarker of disease activity in a platelet of inflammatory disorders, such as vasculitis and atherosclerosis, alongside immunity alterations [133,134,135].

With cancer, previous studies on lung cancer have confirmed that patients produce PTX3 and that there are differential expressions of PTX3 amongst the subtypes of lung cancer with significantly higher levels in small cell lung cancer (SCLC) than non-small cell lung cancer (NSCLC) [136,137]. Collective research also supports the role of PTX3 as a biomarker for the diagnosis of lung cancer, as well as the correlating effect of its expression in tumor proliferation and invasion [138,139]

**Figure 1:**
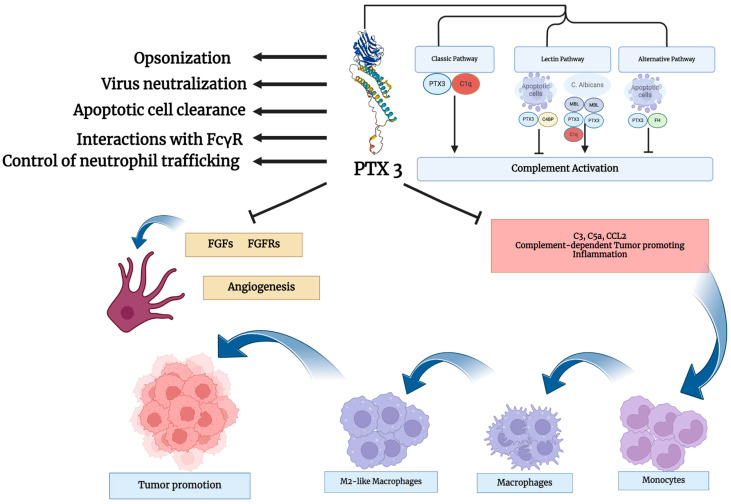
Mechanistic pathways showing the onco-suppressive role of PTX3.

In recent years, PTX3 has also gained great attention in the complex scenario of brain tumors, including GB, due to its pivotal role in innate immunity and inflammatory response [36,66,140]. Specifically, PTX3 has been studied to be a key component of GB microenvironment [35], suggesting its possible role as a biomarker for GB. The onco-suppressive role of PTX3 is illustrated in the Figure 1.

## 5. Influence of Pentraxin 3 in GB

As an essential regulator of the immune system and inflammation, the glycoprotein PTX3 has an emerging critical role in brain tumors. In recent years, studies have demonstrated the involvement of PTX3 in GB growth, suggesting its ability to predict survival outcomes [31,35,141].

Since 2011 and 2013, several researchers have investigated PTX3 expression in samples from patients with gliomas [52,142]. Locatelli and colleagues demonstrated that PTX3 expression differed between low- and high-grade tumors based on histopathological diagnosis and clinical severity [52]. In this regard, the authors discovered higher levels of PTX3 in GB than in low-grade astrocytomas and oligodendrogliomas. In GB samples, PTX3 immunoreactivity was stronger in the cells nearest to the vessel network or in the cells surrounding the necrotic areas, suggesting that PTX3 is a key component of GB microenvironment, being produced by tumor cells and infiltrating CD68-positive macrophages, which are commonly associated with high-grade of gliomas [52].

Afterwards, these results were confirmed in another study performed by Tung et al. [34].

Zhang and colleagues [36] also contributed to the knowledge of PTX3’s role in GB. The authors discovered that, in the context of glioma, PTX3 can recruit multiple immune infiltrating cells and stromal cells, including CD4^+^/CD8^+^ T cells, NK cells, macrophages, fibroblasts, and endothelial cells, suggesting that high expression of PTX3 may promote T reg differentiation and inhibit the proliferation of T cells [36]. Increased expression of PTX3 was negatively correlated with B cell activation, proliferation, and the expression level of IgG, which indicated inhibited humoral immunity, confirming the role of PTX3 in regulating immune infiltrating cells in glioma [36].

In the context of the immune response, the expression of PTX3 in DCs is linked to the humoral immune function of DCs, as a part of soluble pattern recognition molecules, contributes to the recognition of pathogenic microorganisms, as well as the activation of the immune system [140]. Cheng and colleagues [140] discovered that the transcription factor ZNF148 promotes the malignant transformation of DCs after crosstalk with GSCs by upregulating PTX3 and reducing the expression of co-stimulatory factors on the surface of DCs, inhibiting their antigen presentation and T cells activation.

PTX3 is well-known to promote cell proliferation, angiogenesis, and tumor invasion in a variety of cancers, including breast cancer [29], prostate cancer [143], liposarcoma [144], pancreatic cancer [145], and hepatocellular carcinoma [6]. In this regard, it has been demonstrated that PTX3 knockdown in GB cells inhibits proliferation, increases p21 protein levels, and decreases cyclin D1 protein levels, resulting in cell cycle arrest at the G_0_/G_1_ phase [34]. A correlation between high levels of PTX3 and GB cells migration and invasion have also been identified by modulating matrix metalloproteinase-1 and -2 (MMP-1 and MMP-2) expression [34].

Furthermore, a recent study demonstrated that high PTX3 expression in U87 GB cells is associated with angiogenic potential and increased cell migration [66]. In accordance with this, Wesley and colleagues demonstrated that PTX3 promotes tumor growth, angiogenesis, and invasiveness by upregulating VEGF and IL-8 through NF-κB activation, hypothesizing that PTX3 overexpression contributes to GB progression through altered expression of downstream targets involved in angiogenesis, invasion, and migration processes [66].

Interestingly, PTX3 has also been identified as a downstream target by which the spen paralogue and orthologue C-terminal (*SPOC*) domain-containing 1 (*SPOCD1*) genes exert their cancerous effects in gliomas [146]. In this context, Liu and colleagues [146] discovered that SPOCD1 positively regulates the expression of PTX3 in GB, promoting tumor growth.

The role of PTX3 in the process of autophagy in GB has also been explored [141]. Wang and colleagues [141] identified a positive correlation between PTX3 expression and JUN, a transcription factor that encodes the component of the activator protein-1 (AP-1) complex involved in numerous cell activities, including tumorigenesis. This study suggested that JUN participates in cell autophagy modulation by regulating PTX3 expression, promoting tumor progression [141].

These findings reveal the importance of PTX3 in GB development. However, further studies are indispensable to better understand its role in the complex scenario of GB.

The studies cited in the paragraph are shown in Table 1.

## 6. Current Clinical Application of PTX3 Targeting Drugs and Future Perspectives

In the literature, PTX3, as a therapeutic target in clinical trials, received minimal attention. The study conducted by Di Caro and colleagues elucidated how high levels of PTX3 were associated with an increased risk of disease recurrence in 69 patients with colorectal cancer, also advising a direct correlation between PTX3 and worse survival [147]. Similarly, the inflammatory course associated with PTX3 levels has also been evaluated in other types of cancer such as prostate cancer [148] and pancreatic cancer [149].

PTX3 positions as an effector molecule participatory in innate immunity, confirming its immunotherapeutic possibility as a diagnostic and prognostic marker for the improvement of GB treatment. Across all the reviewed previous works on existing malignancies, immunotherapy has demonstrated prognostic feasibilities, and with GB, its efficacy in clinical trials needs to be investigated. The core limitations for immunotherapeutic efforts happen to be the tumor microenvironment being a hostile environment for antitumor responses and the complexities with drug delivery due to the BBB, the high tumor heterogeneity within GB, and chronic toxicities that occur as effects from immunotherapy treatments, which are still valid concerns with no largely successful clinical results so far.

This is what makes our work with PTX3 important. Understanding the mechanistic and molecular pathways in which PTX3 is involved can help develop new targeted therapeutic strategies that address the immunomodulatory concerns with the GB tumor microenvironment.

Due to the limitations of drug delivery and disappointing clinical trials, electric fields with low intensity and intermediate frequency are currently being exploited using electrophoretic drug delivery devices where electric charges are used to transport charged drugs; the device is mounted on the shaved scalp of the patient, and the electric fields alter further tumor development [150,151]. Even though there are limited side-effects of using electric fields, the cost implication is still high. Ultrasound approaches are being developed to help in disrupting the BBB, alongside phase-changed nano droplets specifically designed to carry lipophilic drugs [150]. There are huge positive expectations that the combination of these drug delivery strategies together with immunotherapeutic treatments that are based on biomarkers like PTX3 can overcome the BBB and tumor heterogeneity issue of GB.

In the future, we are certain that synergistic treatment stand the most chance to improve GB treatment and survival, and PTX3 targeting can have a relevant part in this.

## 7. Conclusions

Although numerous studies have been conducted to research new therapies, the survival rate of GB patients remains very low due to the resistance mechanisms exerted by cancerous cells. Considering that tumors, including GB, arise in part from the failure of immunosurveillance, immunotherapy has gained great interest in recent years.

Many researchers have focused on the role of PTX3, an effector molecule belonging to the humoral arm of innate immunity, in GB development. These studies have demonstrated an increase of PTX3 expression in GB patients and its ability to modulate several immune-infiltrating cells, including CD4^+^/CD8^+^ T cells, NK cells, macrophages, and DCs in the glioma microenvironment, suggesting its pivotal role. Additionally, PTX3 has also been widely studied in association with cell proliferation, autophagy, and invasion processes in GB. Therefore, based on these findings, PTX3 could be considered a potential biomarker for GB. Further studies are needed to better understand the mechanisms and molecular pathways in which PTX3 is involved in malignant brain tumors, including GB, to develop new targeted therapeutic strategies.

## Figures and Tables

**Table 1:** Summary of the studies described in this review evaluating PTX3 in GB.

Title of the Paper	Cancer Type	Conclusions of the Study	Reference
Pro-inflammatory gene expression in solid glioblastoma microenvironment and in hypoxic stem cells from human glioblastoma.	GB	PTX3 is expressed in GB.	[142]
The long pentraxin PTX3 as a correlate of cancer-related inflammation and prognosis of malignancy in gliomas.	GB, Astrocytomas, Oligodendrogliomas	PTX3 is a key component of GB microenvironment.	[35]
Inhibition of pentraxin 3 in glioma cells impairs proliferation and invasion in vitro and in vivo.	GB	PTX3 knockdown inhibits GB cell proliferation.	[34]
Pentraxin 3 Promotes Glioblastoma Progression using Negative Regulating Cells Autophagy.	GB	JUN participates in cell autophagy process by regulating PTX3 expression, promoting tumor progression.	[141]
PTX3 mediates the infiltration, migration, and inflammation-resolving-polarization of macrophages in glioblastoma.	GB	PTX3 recruits immune infiltrating cells and stromal cells in GB (CD4^+^/CD8^+^ T cells, NK cells, macrophages).	[36]
Enhanced expression of pentraxin-3 in glioblastoma cells correlates with increased invasion and IL8-VEGF signaling axis.	GB	PTX3 stimulates tumor growth and invasiveness by modulating IL-8 and VEGF.	[66]
Upregulation of the ZNF148/PTX3 axis promotes malignant transformation of dendritic cells in glioma stem-like cells microenvironment.	GB	ZNF148 promotes the malignant transformation of DCs after crosstalk with GSCs by upregulating PTX3.	[140]
SPOCD1 promotes the proliferation and metastasis of glioma cells by up-regulating PTX3.	GB	SPOCD1 contributes to glioma proliferation and metastasis by PTX3 modulating.	[146]

## Abbreviations

GB	Glioblastoma
PTX3	Pentraxin 3
ECM	Extracellular matrix
CRP	C-reactive protein
BBB	Blood–brain barrier
CNS	Central Nervous System
PD-1	Programmed Cell Death 1
PD-L1	Programmed Cell Death Ligand 1
CTLA-4	Cytotoxic T-lymphocyte associated protein 4
PRM	Pattern recognition molecules
NPTX	Neuronal pentraxin
SAP	Serum amyloid P component
HAVCR2	Hepatitis A virus cellular receptor 2
TME	Tumor Microenvironment
TMZ	Temozolomide
TAMs	Tumor-associated macrophages
HGF	Hepatocyte growth factor
VEGF	Vascular endothelial growth factor
TNF	Tumor necrosis factor
APC	Antigen-presenting cells
IL-6	Interleukin 6
CARs	Chimeric antigen receptors
DCs	Dendritic cells
PRRs	Pattern-recognition receptors
MAIT	Mucosa-associated invariant T
NKT	Natural killer T cells
MDSCs	Myeloid-derived suppressor cells
TANs	Tumor-associated neutrophils
CAFs	Cancer-associated fibroblasts
CTL	Cytotoxic T lymphocytes
TCR	T cell receptor
MMP	Matrix metalloproteinase
ICI	Immunostimulatory checkpoint inhibitors
